# Atypical Right Hemisphere Specialization for Object Representations in an Adolescent with Specific Language Impairment

**DOI:** 10.3389/fnhum.2014.00082

**Published:** 2014-02-14

**Authors:** Timothy T. Brown, Matthew Erhart, Daniel Avesar, Anders M. Dale, Eric Halgren, Julia L. Evans

**Affiliations:** ^1^Multimodal Imaging Laboratory, University of California San Diego, La Jolla, CA, USA; ^2^Department of Neurosciences, School of Medicine, University of California San Diego, La Jolla, CA, USA; ^3^Center for Human Development, University of California San Diego, La Jolla, CA, USA; ^4^Department of Radiology, School of Medicine, University of California San Diego, La Jolla, CA, USA; ^5^Program in Experimental and Molecular Medicine, Dartmouth Medical School, Hanover, NH, USA; ^6^Center for Research in Language, University of California San Diego, La Jolla, CA, USA; ^7^School of Behavioral and Brain Sciences, University of Texas Dallas, Dallas, TX, USA

**Keywords:** magnetoencephalography, specific language impairment, object concepts, semantic representations, hemispheric specialization, cerebral dominance

## Abstract

Individuals with a diagnosis of specific language impairment (SLI) show abnormal spoken language occurring alongside normal non-verbal abilities. Behaviorally, people with SLI exhibit diverse profiles of impairment involving phonological, grammatical, syntactic, and semantic aspects of language. In this study, we used a multimodal neuroimaging technique called anatomically constrained magnetoencephalography (aMEG) to measure the dynamic functional brain organization of an adolescent with SLI. Using single-subject statistical maps of cortical activity, we compared this patient to a sibling and to a cohort of typically developing subjects during the performance of tasks designed to evoke semantic representations of concrete objects. Localized patterns of brain activity within the language impaired patient showed marked differences from the typical functional organization, with significant engagement of right hemisphere heteromodal cortical regions generally homotopic to the left hemisphere areas that usually show the greatest activity for such tasks. Functional neuroanatomical differences were evident at early sensoriperceptual processing stages and continued through later cognitive stages, observed specifically at latencies typically associated with semantic encoding operations. Our findings show with real-time temporal specificity evidence for an atypical right hemisphere specialization for the representation of concrete entities, independent of verbal motor demands. More broadly, our results demonstrate the feasibility and potential utility of using aMEG to characterize individual patient differences in the dynamic functional organization of the brain.

## Introduction

Children with receptive or expressive language impairments who have normal hearing, an ordinary environment and rearing experiences, and show no other signs of developmental or neurological disorder are diagnosed with specific language impairment (SLI). Previously referred to as developmental aphasia or dysphasia, SLI is commonly encountered by speech-language clinicians and is found in disproportionate numbers of programs for children and adolescents with academic and behavioral dysfunction (Stark et al., 1988). The psycholinguistic manifestation of SLI can be highly variable across individuals, with primary impairments often evident within multiple aspects of language involving phonology, grammar, syntax, and semantics (Bishop, 2006).

Neurological and cognitive neuroscientific studies of functional brain organization demonstrate a prominent role of the left cerebral hemisphere generally in receptive and expressive language as well as specifically for the representation of semantic information, including word meanings and object concepts (Vigneau et al., 2006). A large body of research demonstrates that semantic knowledge about concrete entities is represented by distributed networks of discrete cortical regions most prominently involving large portions of the left temporal lobe and left ventral prefrontal cortex, as well as parietal and occipital areas (Martin and Chao, 2001; Binder and Desai, 2011), with these regions playing dissociable roles in relatively more perceptual versus conceptual processing. Despite undergoing significant developmental changes (Schlaggar et al., 2002; Brown et al., 2005; Szaflarski et al., 2006) and commonly involving regions of the right hemisphere as well (Martin and Chao, 2001; Binder and Desai, 2011; Donnelly et al., 2011), the typically developing cerebral functional organization for encoding word and object meanings shows a left hemisphere prominence (Martin, 1999) that is present even during infancy (Travis et al., 2011).

Atypical hemispheric specialization in SLI has been suggested in the scientific literature since the early twentieth century (Orton, 1925), but evidence has been inconsistent, particularly within functional neuroimaging experiments (Whitehouse and Bishop, 2008). Volumetric postmortem and structural imaging studies generally have found right-greater-than-left asymmetries in temporal and inferior prefrontal regions in SLI (Jernigan et al., 1991; Plante et al., 1991; Gauger et al., 1997; De Fossé et al., 2004) and greater overall “right-heavy” asymmetry in higher-order association cortex in children with developmental language disorder as compared to the “left-heavy” profile typically shown by control children (Herbert et al., 2005). Pars triangularis (Broca’s area) and perisylvian regions have been implicated specifically, found to be significantly smaller in SLI on the left or to show significantly greater rightward asymmetry (Gauger et al., 1997). At least one structural imaging study found no discernable differences between language impaired and typically developing children in unilateral measurements or bilateral left–right asymmetry of posterior intrasylvian anatomy (Preis et al., 1998).

Functional neuroimaging studies of language impairment have found evidence strongly suggestive of abnormal lateralization patterns in brain activity, but common methodological caveats have often limited a strong interpretation of the results. For example, Whitehouse and Bishop used functional transcranial Doppler ultrasonography (fTCD) to measure cerebral blood flow in 11 young adults with SLI during performance of a letter fluency task (Whitehouse and Bishop, 2008). Interestingly, they compared these subjects with young adults who had a childhood history of SLI but no longer met diagnostic criteria, as well as with adults with a diagnosis of autism and a control group. While silently generating words to a given letter, all of the participants in the SLI-history group and the majority of both the autistic and control subjects showed greater activation in the left compared to the right middle cerebral artery, interpreted by the authors as indicating left hemisphere dominance. In contrast, the majority of individuals with SLI showed brain activity that was deemed either strongly right-lateralized (54.5%) or bilaterally prominent (27.3%).

Atypical hemispheric specialization in SLI has also been suggested in the limited number of functional magnetic resonance imaging (fMRI) studies conducted to date (Hugdahl et al., 2004; Ellis Weismer et al., 2005; Dibbets et al., 2006; Badcock et al., 2012). For example, Badcock et al. compared structural and functional MRI measures during a language task in a group of eight individuals with SLI, their unaffected siblings, and typically developing controls. Anatomically, language impaired participants showed significantly more gray matter than controls in the left inferior frontal gyrus (IFG) and significantly less gray matter in bilateral superior temporal sulcus (STS) and in the right caudate nucleus. Physiologically, when activity during the performance of a covert naming task was contrasted with a silent baseline or passive listening to reversed speech, individuals with SLI showed reduced activity in comparison with the sibling and typical groups. Interestingly, these decrements in brain activity were localized to the same areas implicated in the structural morphological analysis. Furthermore, they observed “clearly left” lateralization of brain activity within the sibling and typical groups, but this was subjectively reduced in SLI. Brain-wide, there were no regions found that showed greater activation in SLI than the other groups. Had this been found, the authors state that it “might have been interpreted as evidence for different functional organization for language or compensatory or maladaptive reorganization.” Badcock et al. also reported that patterns of brain activity in the SLI group were found to show more variability than the unaffected siblings and control group, as measured by laterality indices.

Despite the highly suggestive findings from these fTCD and fMRI studies, covert language tasks were used in these experiments, so no objective measures of subject task compliance and level of performance could be collected during scanning. Therefore, the greater heterogeneity in brain activity and overall under-activation by SLI could be explained simply by worse task performance within the clinical group (Murphy and Garavan, 2004), which would be expected for such tasks based on their diagnosis. Because of this, even for the sibling and control groups used for comparison, there is no way to be reasonably sure that the observed brain activity maps reflect physiological responses that were constrained to the cognitive processes of interest. This issue is common in developmental functional neuroimaging studies and limits the degree to which the desired conclusions can be drawn about the observed differences in functional brain organization (Brown et al., 2003, 2006; Palmer et al., 2004; Poldrack, 2010).

As many of the studies reviewed above point out, atypical cerebral dominance is not evident in all cases of poor language development, nor in all individuals with a diagnosis of SLI. Such patient heterogeneity in functional brain organization can contribute to equivocal results when comparisons are made between a clinical group and control group. Notably, group-averaged brain activity maps reveal only those functional neuroanatomical components that are most similar across subjects and will obscure individual differences. In groups that have especially high inter-individual variability, averaged activity patterns may not be particularly representative of any individual. So, in order to achieve a clearer understanding of the relationships between cognitive functioning and functional brain organization, it may be useful to look more closely at individual patients, particularly with clinical groups such as SLI, which already have been shown to be cognitively and neurologically heterogeneous.

In addition, the majority of functional neuroimaging studies of SLI to date have used fMRI. Despite excellent spatial resolution, fMRI measures neural activity only indirectly, relying on a sluggish vascular response with poor temporal resolution. This inability to separate brain responses in time makes it considerably more difficult to isolate and identify specific cognitive functions that may be driving language task performance, such as sensory, perceptual, semantic, and motor processes (Posner, 1978, 2005; Cohen, 2011).

The purpose of the current study was to use a multimodal neuroimaging technique called anatomically constrained magnetoencephalography (aMEG) to localize with millisecond temporal sensitivity potentially atypical components of the functional brain organization within an individual patient. Here, we used a task paradigm designed specifically to engage cortical systems involved in the semantic representation of concrete objects in an individual with a diagnosis of SLI, comparing him to a group of typically developing individuals with no history of language problems. For comparison, we also applied identical methods to measure the dynamic functional brain organization of this patient’s younger sister, who shows normal language abilities. Although differing by sex and age, she provides a useful comparison of the single-subject analysis methods.

For several reasons, we believed aMEG methods would provide a fruitful approach to the study of one patient. In addition to its sub-millisecond temporal resolution, aMEG provides excellent signal-to-noise properties and enhanced localization of brain activity through the use of noise-normalized source estimates constrained to the cortical reconstruction of each individual subject and aligned using sulcal and gyral surface-based registration (Dale et al., 2000; Dale and Halgren, 2001). Unlike single-dipole fitting MEG methods, the aMEG technique assumes multiple, distributed, and simultaneous cortical generators, which functional neuroimaging and recording methods overwhelmingly show is an appropriate assumption for cognitive tasks.

The primary questions posed in our experiment were: (1) does the dynamic functional brain organization for the semantic processing of concrete objects within an individual with a diagnosis and developmental history of SLI differ from that of a sample of typically developing individuals? (2) If so, how does the functional organization differ, topographically and temporally? (3) Specifically, does this individual show atypical aspects of the functional organization only during latencies that are associated with semantic encoding processes, or does he differ across all latencies measured? (4) Using the same conceptual and methodological approach, does the dynamic functional brain organization of an adolescent sibling with no history of language learning disorder mirror any of the differences found in the individual with SLI, or, instead, appear normal according to these methods? And more generally, (5) do aMEG techniques show feasibility and utility for mapping brain activity within individual patients, using activity distributions from a group of control subjects for direct comparison? In attempting to answer these questions, we hoped to assess both practical and substantive aspects of using aMEG to study individual differences in the dynamic functional organization of the brain.

## Materials and Methods

### Subjects

One left-handed adolescent male diagnosed with specific language impairment (SLI-1; aged 17.8 years), 1 right-handed female sibling (Sib-1; aged 16.1 years), and a group of 12 typically developing right-handed individuals (mean age = 20.9 years, SD = 1.7, range = 18.2–23.5; five female) performed semantic processing tasks during MEG recording. The two individuals who were minors gave assent to participate with parental informed consent, and all control subjects gave informed consent using protocols approved by the UCSD Human Research Protections Program.

The primary characteristic of SLI is the failure to master spoken and written language expression and comprehension despite normal non-verbal intelligence, normal hearing acuity, and no overt physical causes, recognized syndromes, or mitigating medical factors known to cause language disorders in children. SLI-1 was diagnosed at age 4 years with expressive and receptive language delay, meeting criteria for SLI. He had no hearing or other sensory impairments and no history of serious medical problems. He has been followed clinically continuously since that time and received services during school age for language and auditory processing deficits – the only member of his family to qualify for such services. He was never diagnosed with a speech articulation disorder and so did not receive any speech-motor therapy, nor any other special services related to learning and development.

In late adolescence, SLI-1 continues to meet criteria for language impairment. Standard scores (population mean = 100, SD = 15) showed a squarely average non-verbal IQ (102, Leiter-R; Roid and Miller, 1997) but below average performance on a language battery emphasizing grammar and semantics (82; Comprehensive Receptive and Expressive Vocabulary Test, Second Edition – CREVT-2; Wallace and Hammill, 2002). His receptive language score was 82 and expressive language score was 73. Measures of comprehension of non-literal language, deriving meaning from context, and composite language knowledge ranged from about 2 to 2.5 SD below average (72, 60, and 62, respectively; Comprehensive Assessment of Spoken Language – CASL; Carrow-Woolfolk, 1999). On the Clinical Evaluation of Language Fundamentals (CELF-4; Semel et al., 2003), SLI-1 also scored within the clinical range on tests of formulating sentences and recalling sentences (five and six, respectively; mean/SD = 10/3) but scored in the low average range on word classes (eight). On a measure of hand preference based on the Edinburgh questionnaire (Oldfield, 1971), SLI-1 reported being strongly left-handed.

On the same battery of measures, Sib-1 showed above-average non-verbal IQ (127) and above-average performance on a comprehensive language battery emphasizing semantics and grammar (120; CREVT-2). Her receptive language was 1 SD above average (115), and expressive language was within the average range (108). On standardized measures of the comprehension of non-literal meaning, deriving meaning from context, and composite language knowledge, Sib-1 scored within the average range (105, 93, and 99, respectively; CASL). On the CELF-4, she scored within the average to above-average range on tests of formulating sentences, recalling sentences, and word classes (13, 11, and 14, respectively). She reported being strongly right-handed.

Typically developing control subjects were screened by interview and questionnaire to rule out history of developmental learning disorder, head injury, neurological or psychiatric disorder, or other major medical problems. Of the 12 control participants, 8 were undergraduate college or junior college students, and 4 were working full time. All were strongly right-handed. All denied currently taking psychotropic medication. All participants were screened for MRI and MEG safety by self-report and metal detector.

### MEG data acquisition and task paradigm

Event-related fields were measured using a 306-channel whole-head Elekta NEUROMAG system inside a six-layer combination active–passive shielded room (IMEDCO-AG, Switzerland). The tasks, based on previously published studies of semantic processing (Dale et al., 2000; Marinkovic et al., 2003) were pilot-tested and modified to be more easily performed by children and adolescents and involved the presentation of two types of visual stimuli: printed words (high frequency, early acquired, highly imaginable concrete nouns; e.g., bed, mouse, door, whale, bug, house, leaf) and simple line drawings of lexically equivalent common, nameable objects. Picture and word stimuli were presented as white lines or letters against a black background. Subjects were instructed to respond to each item with laser-detected index finger lifts (one with the left hand, one with the right), indicating whether or not the object conveyed by word or image was small enough in size to fit into a shoebox. Stimuli were balanced with regard to the number of large versus small objects. Onscreen stimulus duration for words and pictures was 300 ms, delivered with interstimulus intervals jittered between 3 and 5 s. Words and pictures were presented roughly equal in size, subtending approximately 4° of visual angle in their largest dimension. Picture and word stimuli were delivered in eight separate, alternating task runs (four picture runs, four word runs), each presenting 40 items and lasting about 4 min.

### MRI data acquisition

High-resolution T1-weighted structural MRI scans optimized for gray/white matter contrast were acquired at 1.5 T for all subjects [time to echo (TE) = 3.8 ms, time to repetition (TR) = 10.7 ms, time to inversion (TI) = 1000 ms, flip angle = 8°, trigger delay (TD) = 750 ms, bandwidth = 31.25 Hz/pixel, field of view (FOV) = 24 cm, matrix = 192 × 192, slice thickness = 1.2 mm]. Real-time head motion tracking and correction was performed using PROMO, as described previously for prospective motion correction in spiral-navigated 3D pulse sequences (White et al., 2010). PROMO has been shown qualitatively and quantitatively to significantly improve image quality and reduce distortions caused by head motion and related artifacts, to increase the reliability of MR-derived brain measures (e.g., volume, thickness), and to improve the clinical diagnostic utility of structural MRI data when acquired in difficult-to-scan groups such as children (Brown et al., 2010; Kuperman et al., 2011).

### Multimodal image processing and analysis

Multimodal brain activity maps were produced by generating a three-dimensional reconstruction of the cortical surface for each individual using MRI data (Fischl et al., 1998, 1999a,b; Dale et al., 1999) and spatially constraining the source estimations of MEG-derived noise-normalized dipole strength to its geometry (Dale et al., 2000; Dale and Halgren, 2001). Dynamic statistical parametric maps (dSPMs) of cortical activity were computed for the two individuals and for the average of the typically developing group, spanning seven time windows based on previous aMEG studies of visual lexical semantic processing (Dale et al., 2000; Marinkovic et al., 2003; Leonard et al., 2010, 2011). Time windows were chosen to display separable brain activity events occurring across sensory, perceptual, and cognitive-semantic processing stages. At early latencies, beginning at 120 ms, windows 50 ms wide were used to reveal brief, localized visual activity that has been observed in previous aMEG studies using visual semantic paradigms with similarly timed events (Dale et al., 2000; Marinkovic et al., 2003; Leonard et al., 2010, 2011). Rather than choosing single dSPM frames at several discrete latencies (that is, show maps restricted to exactly one selected time point, such as 120, 158, or 400 ms), data were averaged within these time window blocks and displayed as such to provide a more complete representation of total brain activity over time, and one less prone to frame selection biases. dSPMs were produced using only trials with correct task responses, reflecting cortical activity only during the successful evocation of semantic representations in every participant.

In order to more directly reveal how the dynamic patterns of cortical activity in SLI-1 and Sib-1 probabilistically compare with that of control subjects, and to avoid relying solely on qualitative evaluations of thresholded dSPM images, we also computed *z*-statistic maps showing the degree of similarity and dissimilarity in activity amplitude estimates at all cortical locations and all time points for both SLI-1 and Sib-1 in relation to the distribution of cortical activity for the comparison group, expressed in standard deviation (SD) units.

## Results

### Behavior

During MEG recording, behavioral task accuracies were similar between the two siblings for both word (SLI-1 = 68%; Sib-1 = 69%) and picture stimuli (SLI-1 = 83%; Sib-1 = 80%). Out of 160 total words, SLI-1 responded accurately to 109 items, and SIB-1 110. For pictures, SLI-1 responded correctly to 133 stimuli, and SIB-1 128 items. Response times (RTs) were somewhat slower for SLI-1 for both words [SLI-1 = 1044 ms (mean)/438 (SD); Sib-1 = 915/548] and pictures (SLI-1 = 877/269; Sib-1 = 766/302).

On average, typically developing subjects performed more accurately and faster than the siblings for both words (89/6%, range = 77–96; RT = 820/146, range = 590–1063) and pictures (87/7%, range = 75–96; RT = 757/125, range = 582–957). Out of 160 words total, the control group responded correctly on average to 142 items and ranged across individuals from 123 items correct to 154. For picture stimuli, the mean number of items correct for the control group was 139 out of 160, with a range from 120 correct trials to 154. Therefore, for word stimuli, both of the siblings performed with lower accuracy than the lowest performing control subject. For pictures, however, they both outperformed the lowest scoring control participant. As indicated by the RT ranges, both SLI-1 and Sib-1 responded, on average, faster for both words and pictures than the slowest typically developing control subject.

### Imaging

**Figure 1:**
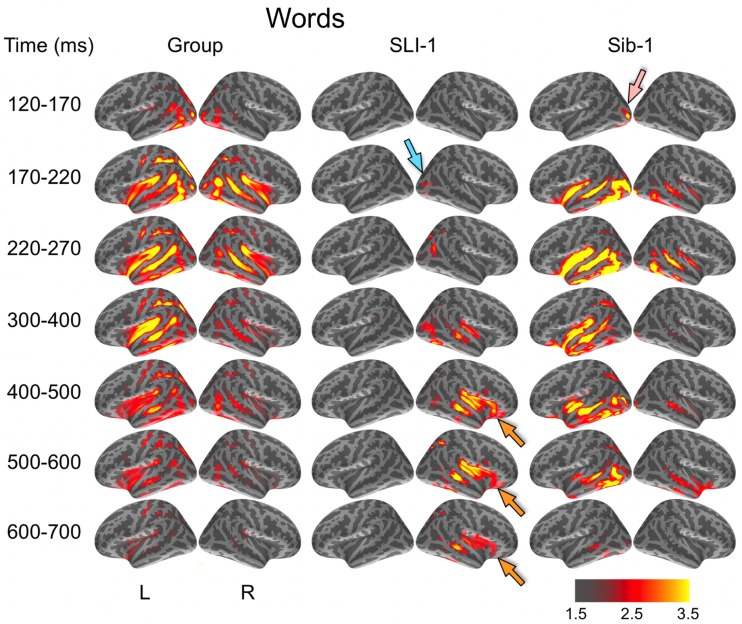
**Group and single-subject dSPMs of mean cortical activity during the semantic processing of words**. In comparison to the functional organization of both the control group and sibling, SLI-1 showed strongly right-lateralized activity, from early sensoriperceptual to later cognitive stages. His early lateral occipital response was on the opposite side and somewhat delayed in time (blue arrow) in relation to his sister (pink arrow). During latencies typically associated with semantic encoding, he showed sustained activity within right temporal, perisylvian, and frontal opercular regions (orange arrows). Color scale represents square root of *F* values, which are a measure of signal-to-noise.

Noise-normalized dSPMs for the typically developing control group were strongly consistent with the results of previous aMEG and fMRI studies of the semantic processing of words and pictures (Dale et al., 2000; Dale and Halgren, 2001; Martin and Chao, 2001; Marinkovic et al., 2003; Vigneau et al., 2006; Leonard et al., 2010, 2011; Binder and Desai, 2011). During the processing of words, early lateral visual responses occurred between 120 and 170 ms in bilateral occipitotemporal regions and were stronger on the left (Figure 1). Within 50 ms, activity spread across multiple regions bilaterally, including intraparietal and transverse occipital sulci, lateral occipitotemporal and temporal cortex, and anteriorly along perisylvian regions. By 300 ms, cortical activity became more strongly left lateralized and included left frontal operculum after about 400 ms. Qualitatively, Sib-1 showed a dynamic functional brain organization for processing words that was similar to the typically developing group. Her earliest lateral visual response occurred during 120–170 ms and was located in left middle occipital sulcus (pink arrow). Activity then spread bilaterally and anteriorly along occipitotemporal and perisylvian regions and, similar to the group, became more strongly left lateralized at 300 ms. Sustained left lateralized activity was apparent through 600 ms and at 500 ms included bilateral anterior insula and temporal poles.

In striking contrast to both the comparison group and to his younger sister, SLI-1 showed no cortical activity within the left hemisphere that surpassed the same threshold during the semantic processing of words. In general, his functional neuroanatomy was notable for being strongly right-lateralized, less distributed, and somewhat delayed in time. In contrast to the other subjects, the first discernable lateral visual response for SLI-1 occurred at 170–220 ms and was located within the right hemisphere (blue arrow). Activity then spread anteriorly more slowly and only on the right, engaging middle temporal, and right perisylvian regions only by about 300 ms. From 400 to 700 ms, SLI-1 showed sustained activity within right middle temporal, perisylvian, and frontal opercular areas (orange arrows).

**Figure 2:**
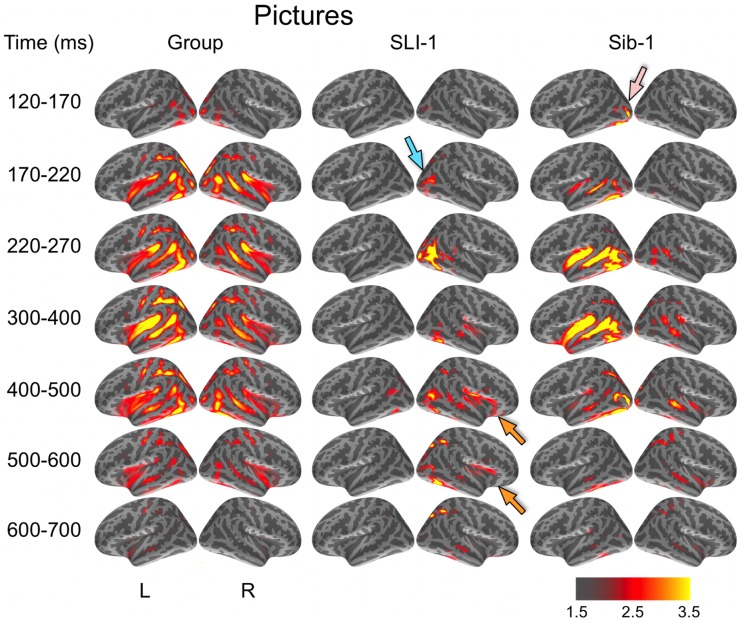
**Group and single-subject dSPMs of mean cortical activity during the semantic processing of pictures**. In comparison to the functional organization of both the control group and sibling, SLI-1 showed strongly right-lateralized activity, from early sensoriperceptual to later cognitive stages. Similar to word processing, his early lateral occipital response was on the opposite side and somewhat delayed in time (blue arrow) in relation to his sister (pink arrow). During latencies typically associated with semantic encoding, he showed activity within right temporal, perisylvian, and frontal opercular regions (orange arrows), although weaker than for words. Color scale represents square root of *F* values, which are a measure of signal-to-noise.

During the evocation of semantic representations by picture stimuli, typically developing subjects showed spatiotemporal activity patterns that varied from word stimuli in ways consistent with previous aMEG studies. In general, neural activity was less strongly left lateralized, including early lateral visual responses within posterior occipitotemporal regions as well as later, from 300 to 600 ms (Figure 2). As with word processing, the dynamic functional organization shown by Sib-1 for pictures was similar to that of the comparison group, although qualitatively more strongly left lateralized. Her earliest lateral visual response was likewise apparent within the 120- to 170-ms time window, but only within middle and inferior occipital sulci on the left (pink arrow). Activity then spread anteriorly along left occipitotemporal and perisylvian regions and was weaker on the right than for the typically developing controls. From 300 to 500 ms, activity for Sib-1 was somewhat more bilaterally evident for pictures than it was for words. Overall, her engagement of cortical areas during the presentation of pictures declined earlier than for words, especially within left anterior temporal and insular regions.

Just as for word stimuli, SLI-1 showed a functional neuroanatomy during the evocation of object concepts by pictures that were very different from both his sister and the comparison group. Again, his spatiotemporal patterns of activity were most notable for being strongly right-lateralized and somewhat delayed in time. Similar to words, picture stimuli evoked an early lateral visual response at 170–220 ms within right anterior occipital sulcus (blue arrow). Activity then spread first throughout proximal right occipital areas then involved right posterior perisylvian regions weakly. From 400 to 600 ms, SLI-1 showed sustained activity within right perisylvian and frontal opercular regions similar to (but weaker than) that observed during his semantic processing of words (orange arrows).

**Figure 3:**
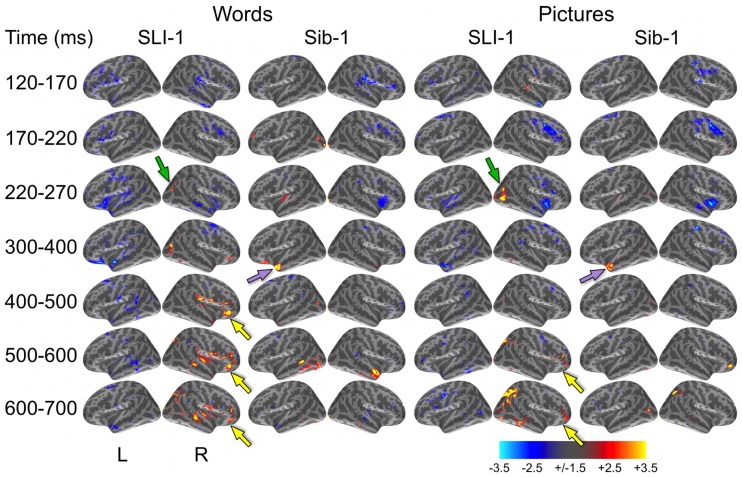
***z*-Statistic maps of single-subject cortical activity in relation to the typically developing control group during the semantic processing of words and pictures**. In direct comparison to the control group, SLI-1 showed relative under-activation of many left hemisphere regions and over-activation of only right hemisphere regions. This included early over-recruitment of right lateral occipital cortex for both words and pictures (green arrows), as well as later over-recruitment of right frontal opercular regions during semantic latencies (yellow arrows). Compared to controls, Sib-1 showed early under-engagement of right anterior regions for words and pictures, consistent over-engagement of left temporal pole 300–400 ms (violet arrows), and over-recruitment of several left and right areas at the latest time windows. Color scale represents *z*-statistics (standard deviation units).

In a direct, vertex-wise comparison to the distribution of neural activity at all cortical locations and time points within the typically developing group using *z*-scores, SLI-1 showed differences from the typical dynamic functional organization that agreed with qualitative comparisons of the dSPMs. During the semantic processing of words, SLI-1 showed relative under-recruitment of many cortical regions bilaterally at early latencies, including perisylvian, anterior temporal, opercular, and lateral and superior frontal cortex (Figure 3). Beginning at 220 ms, he showed the strongest areas of relative under-engagement within left frontal opercular and anterior temporal regions, continuing to 400 ms. At the same time, he began to show notable relative over-recruitment of regions within the right occipital cortex (green arrow), which also extended to 400 ms. At 400 ms, SLI-1 showed greater activity than typically developing controls in several right hemisphere perisylvian cortical areas extending from subcentral sulcus to right frontal operculum. These regions showed sustained relative over-activity that continued from 500 to 700 ms (yellow arrows), where additional right hemisphere temporal and parietal over-activity also became apparent. When picture stimuli were used to evoke object representations, SLI-1 showed only relative under-activation within the left hemisphere and over-activation only within the right hemisphere. Just as for words, he showed early over-recruitment of right middle and inferior occipital regions beginning at 220 ms (green arrows). From 500 to 700 ms, he showed late over-recruitment of right frontal regions similar to those for words (yellow arrows) and also over-recruitment of parietal, occipital, and temporal areas.

At the earliest latencies, the *z*-maps for Sib-1 revealed relative under-engagement of many bilateral anterior regions during word and picture processing that was similar to her brother. Interestingly, however, she showed notable relative over-recruitment of the left temporal pole in relation to the control group, which was consistently at 300–400 ms for both stimulus types (violet arrows). At late semantic processing stages, several cortical regions within right frontal and parietal, bilateral temporal, and left occipitotemporal cortex, showed greater levels of activity than the comparison group for pictures and words.

## Discussion

The primary purpose of our study was to test the feasibility and usefulness of anatomically constrained MEG for making direct comparisons between individual patients and typically developing control subjects in the dynamic functional organization of the brain. Using this multimodal functional neuroimaging technique, which provides the uncommon ability to localize cortical activity with millisecond temporal resolution, our findings revealed in an individual patient with a history of developmental language disorder an atypical right hemisphere specialization for the semantic representation of concrete entities.

Several aspects of our study support this interpretation. Using single-subject statistical maps, this patient showed strongly right-lateralized brain responses during the successful performance of a task that requires the evocation of semantic representations of visual objects when no spoken verbal response was required. Strong engagement of right hemisphere perisylvian regions was observed even during middle and late latencies typically associated with semantic encoding processes. His marked right hemisphere predominance was evident from early sensoriperceptual through later cognitive processing stages and was utilized for the semantic processing of both word and picture stimuli. During performance of the same tasks, typically developing control subjects, in contrast, showed bilateral involvement at early latencies followed by activity predominantly within the left hemisphere, especially during the processing of words. This is strongly consistent with the topography and timing from previous aMEG and fMRI studies of the semantic processing of pictures and words. As an additional control comparison for our single-subject analysis methods, the dynamic functional brain organization of a younger sibling with normal language development was found to be largely similar to the control group. Vertex-wise direct comparison of the patient and sibling to the distribution of cortical activity at every location and time point shown by the control group verified differences revealed by the comparison of independent brain activity maps.

Altogether, the functional neuroanatomical differences for this individual suggest a supramodal neural system for object concepts that appears similar to the left lateralized organization previously observed within association cortex in typically developing adults (Marinkovic et al., 2003), except that it is supported by the right hemisphere and appears to be engaged somewhat later in time. In bypassing reliance on auditory input, avoiding speech-motor demands, recording overt behavioral responses, mapping only successful trials, and localizing brain activity with millisecond temporal resolution, our interpretation of the functional organization can be more convincingly constrained to operations that involve semantic encoding and object representations. In contrast with traditional approaches testing cerebral functional specialization using naming, our findings were obtained without requiring speech-motor production components of language processing.

Language is made possible by a complex set of processing operations involving distributed mechanisms within the brain. Perhaps because of this, its development is surprisingly robust in the face of adverse neurological circumstances, such as early stroke, head trauma, and even hemispherectomy (Muller et al., 1998; Vicari et al., 2000; Bates et al., 2001; Fair et al., 2006; Liegeois et al., 2008; Trauner et al., 2013). This strongly suggests that there are multiple pathways to effective language learning and that the brain finds a detour when one pathway is blocked. Children who receive a clinical diagnosis of SLI, however, tend not to have a single problem cognitive area and instead display multiple underlying deficits (Bishop, 2006). By selectively probing object representations within only one patient, we hoped to learn something about the functional brain organization that might not be apparent from a group-averaged imaging study where patient heterogeneity in functional organization might produce equivocal results. Interestingly, we found strongly right-lateralized cortical responses within the very first individual with SLI we have tested.

Although these results strongly suggest an atypical functional brain organization for semantic processing, our study is limited in providing leverage to make inferences about several important etiological factors. Theories of abnormal right hemisphere involvement in developmental language disorders have existed for decades and emphasize atypical cerebral dominance for the motor control of speech and limb praxis (Zangwill, 1960; Satz, 1972; Geschwind and Galaburda, 1985). So, tasks used to test language lateralization typically employ an overt verbal production component such as spoken naming. However, even among left-handers such as SLI-1, who represent only about 10% of the world’s population (Hardyck and Petrinovich, 1977), estimates of right hemisphere language dominance are thought to be relatively rare, ranging between about 7 and 27% even when mixed/bilateral dominance is included (Rasmussen and Milner, 1977; Knecht et al., 2000; Drane et al., 2012). This means that only somewhere between 0.7 and 2.7% of the general population would be estimated to show right hemisphere or bilateral language dominance for spoken naming, the task commonly used in these studies. The fact that the large majority of left-handed individuals still seem to have left-dominant language representation suggests that it is common for dominant limb motor control to be decoupled from (i.e., contralateral to) dominant language representation.

The implications for the lateralization of the motor control of speech specifically, which is thought to follow lateralization for handedness, are unclear from available evidence. When language lateralization is probed using hemispheric anesthetization (i.e., the Wada procedure), are object concepts consistently represented within the hemisphere predominantly responsible for verbal motor control and speech production? Put another way, is a patient’s inability to name objects in these experiments driven solely by arrest of the verbal articulators, by an inability to access the semantic representations required for naming, or by both? By disentangling these functions within an individual patient with a language learning disorder, we hoped to identify specifically whether object concepts themselves might be functionally organized in an atypical fashion, independent of verbal production demands. Perhaps some forms of developmental language learning disorder are caused by a mismatch between which hemisphere is dominant for speech-motor control and which specializes for the semantic encoding of object concepts and word meanings, causing access difficulties during language production. Although SLI-1 is strongly left-handed, this could be the case for his verbal praxis and will need to be tested further.

Our findings with SLI-1 are consistent with a number of cognitive developmental interpretations and models of hemispheric specialization, including possibly “weaker” semantic representations and the coarse encoding hypothesis (Beeman et al., 1994; Borovsky et al., 2013). His spontaneous task performance level, which provides one objective measure of the strength or accessibility of these semantic representations, might suggest that SLI-1’s representations are no weaker than those of his sister, who performed similarly and nevertheless did not show strongly right-lateralized activity. Within the context of his clinical profile and developmental history, however, the present findings certainly suggest that his atypical functional brain organization is a contributor to and/or a product of his difficulties with language learning.

Interestingly, both SLI-1 and Sib-1 showed regions of sustained relative underactivity as compared to the control group during the processing of both words and pictures. This under-recruitment was most prominent at early latencies, from stimulus onset until about 270 ms, and included perisylvian regions, particularly on the right for Sib-1. These effects may relate to speed of processing differences between these two individuals and the control group. Although both SLI-1 and Sib-1 responded, on average, faster for both words and pictures than the slowest typically developing control subject, their behavioral RTs were nevertheless slower than the average of the comparison group. SLI-1 responded on average 224 ms slower than the average of controls while processing words and 120 ms slower for pictures. Sib-1’s average behavioral response was 95 ms slower than the control average for words but only 9 ms slower than that for control participants for pictures, suggesting that these early decrements in activity within the right hemisphere cannot be solely accounted for by slower task performance.

Several additional substantive issues and limitations with our study warrant further discussion. First, our group of control participants differed from one or both of the other individuals along two relevant characteristics: age and handedness. Because of this, we do not attempt to make any strong inferences about either the specific role of handedness in our findings or about the developmental state or phase of the individual subjects. Since this experiment employed a relatively untested collection of techniques as applied to single patients, we began by comparing SLI-1 to a group representative of typically developing adolescents and young adults comprised of right-handed individuals. Indeed, it would be interesting and informative to compare SLI-1 in the same way to an individual or group showing otherwise cognitively normal left-handedness. Such a comparison would be required to make inferences about the specific role of handedness in SLI-1’s functional neuroanatomical differences. However, the scientific evidence would suggest, assuming a representative subject or sample of left-handed participants was obtained, that a direct comparison of SLI-1 with them might yield results similar to what we found in right-handers. Nevertheless, this is an empirical question that will require future experiments. The present study is only able to address the first-order question of how the dynamic functional brain organization for semantic processing in an individual with SLI differs from typically developing, right-handed controls.

Secondly, the range of ages for the control group was not ideal for making inferences about the two siblings in relation to age-matched peers, since the control group was somewhat older. The individual with SLI was 3.1 years younger than the control group average and 0.4 years younger than the youngest control subject. His sister (Sib-1) was 4.8 years younger than the control group average and 2.1 years younger than the youngest control subject. So, we attempt to make no inferences about either of these individuals in relation to the ages or developmental phase of the control group. Instead, we have characterized brain activity in each participant both independently of the other participants and in direct relation to the distribution of brain activity from the same control group. So, their *z*-stat maps show levels of activity relative to the same distribution, making them a useful comparison with one another despite their age difference. Additionally, since SLI-1 is older than Sib-1, this comparison provides evidence that SLI-1’s atypical functional organization cannot be solely attributed to being somewhat younger than the control sample. If this were true, his even younger sibling should show the same pattern.

That being said, available evidence from large-scale functional neuroimaging studies suggests that data from one 16- or 17-year-old participant would not be developmentally detectably different from that of individuals 18- to 23-years-old, because the developmental signal at these ages will be overpowered by the vast range of differences across individuals, even of the same age. From early school age into young adulthood, the range of individual differences variability in brain activity measures at a given age far exceeds the range of developmental changes that occur on average across even several years of development (Brown et al., 2005; Dosenbach et al., 2010). These studies, as well as positron emission tomography (PET) measurements of cerebral glucose metabolic rates (Chugani et al., 1987; Chugani and Phelps, 1991), also show that the slope of annualized developmental changes in activity decreases from late grade school age into adolescence and young adulthood, asymptoting during the ages studied here. This has been shown to be similar for many anatomical brain features as well, including morphological, diffusion, and signal intensity measures (Giedd et al., 1999; Sowell et al., 2003; Brown et al., 2012). Our data collected from Sib-1 demonstrate this point. Despite being about 2 years younger than the youngest control subject and about 5 years younger than the average age of the control group, the cortical regions that she engages during semantic processing show activity levels that fall predominantly within a similar dynamic range. Nevertheless, the specific ages of the control comparison group will become eminently more consequential when one seeks to make specific *maturational* or *developmental* inferences about the brain activity measures of one individual.

In light of our atypical findings for SLI-1, the data from the younger sibling become an especially useful comparison. Using methods identical to those applied with the language impaired adolescent, including use of the same statistical thresholds, both kinds of maps computed for Sib-1 (i.e., independent thresholded dSPMs and *z*-maps relative to the distributions of brain activity from the control group) revealed a functional brain organization that is largely similar to the control group. This provides evidence that the anomalous nature of the results found with SLI-1 cannot be explained simply by the single-subject analytic approach. The patterns of brain activity in a younger, language-typical individual – even from the same family – show a more typical functional organization for the same tasks.

Fortuitously, the siblings performed similarly on both types of cognitive tasks during MEG recording. This strengthens the confidence with which we can fairly compare their observed cortical functional organizations. Specifically, it further suggests that SLI-1’s strongly rightward organization is not due solely to the fact that he was performing more poorly than the control group on average. If this were the case, Sib-1 would have shown similar right-lateralized activity. This point is important in light of recent MEG studies that have shown that right hemisphere participation in semantic decision tasks may increase with greater task difficulty in adults (Donnelly et al., 2011) or with objects that are never-before-seen and for which the names are newly encoded in children (Urbain et al., 2013). Interestingly, a very similar experimental paradigm using novel objects and names with adults showed instead that learning the names of new objects utilizes a cortical network very similar to the set of regions used for naming familiar items (Cornelissen et al., 2004). Important to note for all of these studies, left hemisphere activity was prominent despite relative increases in right hemisphere involvement. This progression from strong bilateral to reduced right hemisphere involvement during word learning is consistent with findings from studies of normal developmental changes in lexical semantic processing using both fMRI (Schlaggar et al., 2002; Brown et al., 2005; Szaflarski et al., 2006) and MEG (Ressel et al., 2008), as well as for second-language word learning in bilingual adults (Leonard et al., 2010, 2011).

More broadly, our results demonstrate the feasibility and potential utility of using aMEG to characterize individual differences in the cortical activity dynamics associated with specific cognitive functions. Scientifically and clinically, there are many reasons for developing improved functional neuroimaging methods to characterize single patients, not the least of which is to inform diagnostic assessment and individualized treatment planning. However, there have been technical, methodological, and conceptual barriers commonly encountered, such as weak signal-to-noise characteristics of the brain activity measures taken from only one patient, as well as the limited statistical approaches that can be adopted for making probabilistic comparisons and hypothesis tests based on data from a single-subject. Here, we used a whole-brain *z*-statistic technique that has been employed previously in case studies with fMRI data (Turkeltaub et al., 2004; Fair et al., 2006), which has the benefit of being conceptually straightforward but remains statistically descriptive.

Further development of multimodal functional neuroimaging approaches for single patients, such as with aMEG, will be crucial for providing a better understanding of the specific subcomponents underlying atypical brain-cognition-behavior linkages. Future aMEG experiments with both healthy and clinical subjects should focus on a more nuanced relation of the temporal dynamics of the functional brain organization to specific information processing operations, moving away from simple dichotomies involving broad psychological constructs such as left versus right “language dominance.” Further, much work is needed in characterizing how this dynamic functional mosaic changes across different ages. We believe that more research into these kinds of distinctions will help refine our understanding of the role of hemispheric specialization in developmental language disorders and how sensoriperceptual processes, motor control, and semantic representations come together to support human language. This will undoubtedly aid in the early detection of developmental cognitive disorders, biologically inform our clinical diagnostic schemes, and improve our ability to individually tailor treatments.

## Author Contributions

Timothy T. Brown and Eric Halgren designed the experiment. Eric Halgren and Anders M. Dale developed the multimodal imaging methods. Timothy T. Brown, Matthew Erhart, and Daniel Avesar collected the MEG and MRI data, and Julia L. Evans collected and interpreted the behavioral data and performed the clinical diagnosis. Timothy T. Brown, Matthew Erhart, and Daniel Avesar processed and analyzed the imaging data. Timothy T. Brown wrote and edited the manuscript with input from the other authors.

## Conflict of Interest Statement

Anders M. Dale and Eric Halgren are founders of and hold equity interest in Cortechs Labs, La Jolla, CA, USA and serve on its scientific advisory board. The terms of this arrangement have been reviewed and approved by UCSD in accordance with its conflict of interest policies. The other co-authors declare that the research was conducted in the absence of any commercial or financial relationships that could be construed as a potential conflict of interest.
